# Theoretical Study of the C_2_H_5_ + HO_2_ Reaction: Mechanism and Kinetics

**DOI:** 10.3390/molecules23081919

**Published:** 2018-08-01

**Authors:** Nan-Nan Wu, Ming-Zhe Zhang, Shun-Li Ou-Yang, Liang Li

**Affiliations:** 1 Key Laboratory of Integrated Exploitation of Bayan Obo Multi-Metal Resources, Inner Mongolia University of Science &Technology, Baotou 014010, China; woshinannan04@imust.cn (N.-N.W.); imust2016023081@163.com (M.-Z.Z.); 2 School of Science, Inner Mongolia University of Science and Technology, Baotou 014010, China; 3 College of Physics, Jilin University, Changchun 130012, China; lliang@jlu.edu.cn

**Keywords:** C_2_H_5_, HO_2_, mechanism, kinetics

## Abstract

The mechanism and kinetics for the reaction of the HO_2_ radical with the ethyl (C_2_H_5_) radical have been investigated theoretically. The electronic structure information of the potential energy surface (PES) is obtained at the MP2/6-311++G(d,p) level of theory, and the single-point energies are refined by the CCSD(T)/6-311+G(3df,2p) level of theory. The kinetics of the reaction with multiple channels have been studied by applying variational transition-state theory (VTST) and Rice–Ramsperger–Kassel–Marcus (RRKM) theory over wide temperature and pressure ranges (T = 220–3000 K; P = 1 × 10^−4^–100 bar). The calculated results show that the HO_2_ radical can attack C_2_H_5_ via a barrierless addition mechanism to form the energy-rich intermediate **IM1** C_2_H_5_OOH (68.7 kcal/mol) on the singlet PES. The collisional stabilization intermediate **IM1** is the predominant product of the reaction at high pressures and low temperatures, while the bimolecular product **P_1_** C_2_H_5_O + OH becomes the primary product at lower pressures or higher temperatures. At the experimentally measured temperature 293 K and in the whole pressure range, the reaction yields **P_1_** as major product, which is in good agreement with experiment results, and the branching ratios are predicted to change from 0.96 at 1 × 10^−4^ bar to 0.66 at 100 bar. Moreover, the direct H-abstraction product **P_16_** C_2_H_6_ + ^3^O_2_ on the triplet PES is the secondary feasible product with a yield of 0.04 at the collisional limit of 293 K. The present results will be useful to gain deeper insight into the understanding of the kinetics of the C_2_H_5_ + HO_2_ reaction under atmospheric and practical combustion conditions.

## 1. Introduction

With low molecular weight, small polarity, and high volatility, alkanes can easily enter the atmosphere and are one of the main components of urban atmospheric pollutants. They are mainly released into the atmosphere through abstraction, distillation, refining, and combustion of fossil fuel, as well as combustion and natural decomposition of organics. Alkanes mainly make oxidization reactions with free radical –OH to produce an active alkane radical (R∙). R∙ is also the initial product of pyrolysis, oxidization, combustion, or a photochemical reaction of saturated hydrocarbons. Besides, it can make a quick addition reaction with O_2_, an important step to generate an alkane peroxy radical (RO_2_∙). RO_2_∙, the simplest organic peroxy radical, is an important intermediate of combustion and oxidization of hydrocarbons. It is the key to low temperature combustion, flame propagation, and spontaneous combustion of fuels. Considering the actual application values of R∙ in combustion, atmospheric chemistry, and biological process [1,2,3,4,5,6,7,8], many experimental and theoretical research studies on their reactions with H, O and O_2_, as well as their own reactions have been reported. It is common knowledge that the hydrogen peroxide radical (HOO∙) is an important transient intermediate of hydrocarbon fuel combustion, atmospheric photolysis circulation, and biochemical process, and has a considerable high concentration in the troposphere [9]. Hence, R∙+HOO∙ reactions play an important role in the combustion chemistry of hydrocarbons and degradation of alkanes. Additionally, they will affect the balance of R∙ + O_2_ ↔ RO_2_∙significantly and generate a potential deep chain source.

The ethyl radical C_2_H_5_ has the simplest structure and could show many oxidation characteristics of big R∙, including those generated by olefins [2]. Therefore, studying the C_2_H_5_ + HOO reaction is important to establish a model of the R∙+ HOO∙ reaction.

The bimolecular C_2_H_5_ + HOO reaction can occur indirectly by generating an intermediate with rich energy:

C_2_H_5_ + HOO→CH_3_CH_2_OOH * (+M) (1)

C_2_H_5_ + HOO→CH_3_CH_2_O + OH (1a)

C_2_H_5_ + HOO→CH_3_CHO + H_2_O (1b)

C_2_H_5_ + HOO→CH_3_CH_2_OOH (1c)

or through hydrogen abstraction:

C_2_H_5_ + HOO→CH_3_CH_3_ + ^3^O_2_ (1d)

C_2_H_5_ + HOO→CH_3_CH_3_ + ^1^O_2_ (1e)

C_2_H_5_ + HOO→CH_2_=CH_2_ + H_2_O_2_ (1f)

where the * represents internal excitation.

Three experimental groups have studied the C_2_H_5_ + HOO reaction. Tsang and Hampson [10] first studied the kinetics of the C_2_H_5_ + HOO reaction over the temperature range 300–2500 K. They suggested that the generation channel of C_2_H_5_O + OH is the most feasible reaction channel, and estimated the reaction rate constant of this channel *k*_1a_ = 4.98 × 10^−11^ cm^3^·molecule^−1^·s^−1^. They also proposed another two products: C_2_H_4_ + H_2_O_2_ and C_2_H_6_ + O_2_, whose reaction rate constants were estimated as *k*_1f_ = *k*_1e_ = 5.00 × 10^−13^ cm^3^·molecule^−1^·s^−1^. In 1993, Dobis and Benson discovered that the reaction rate constant of C_2_H_4_ + H_2_O_2_ is *k*_1f_ = 2.97 × 10^−12^ cm^3^·molecule^−1^·s^−1^ over the temperature range of 243–368 K [11], but they did not detect the expected major product C_2_H_5_O + OH. Recently, Temps and his partners studied the C_2_H_5_ + HOO reaction by combing time-dependent mass spectrum and laser photolysis/recirculation reactor [3]. They found that the overall reaction rate constant at 293 K is 5.15 (±1.66) × 10^−11^ cm^3^·molecule^−1^·s^−1^. They detected C_2_H_5_O and pointed out that the generation channel of C_2_H_5_O + OH is the major reaction channel. However, no clear experimental result on the mechanism of this complicated reaction with multiple potential wells and multiple channels, pressure and temperature dependence of its products within a wider measuring range, as well as product distribution has been reached yet. As far as we know, there has been no theoretical research on this reaction. Considering the significance of the C_2_H_5_ + HOO reaction in combustion and atmospheric chemistry, it is necessary to make a comprehensive theoretical study on its mechanism and kinetics. In this paper, potential energy surfaces (PES) of the C_2_H_5_ + HOO reaction were explored through quantum chemistry and its kinetics were computed using the Rice–Ramsperger–Kassel–Marcus (RRKM) unimolecular reaction rate theory of microcanonical ensemble [12]. Data of potential energy surfaces are theoretical data. Variations of reaction rate constants and branching ratios of many dissociation products and intermediates with temperature and pressure were discussed. 

## 2. Calculation Methods

The geometries of all of the reactants, products, intermediates, and transition states involved in the C_2_H_5_ + HO_2_ reaction were optimized at the second-order Møller-Plesset perturbation MP2 [13] method in conjunction with the 6-311++G(d,p) basis set. Frequency analysis was performed at the same level to check whether the obtained species is a local minima (with all real frequencies) or a transition state (with only one imaginary frequency). The minimum reaction path (MEP) was calculated by intrinsic reaction coordinate (IRC) [14,15,16,17] to confirm that the transition states connect the designated intermediates. To obtain more reliable energetics, the single-point energies were refined at the CCSD(T)/6-311+G(3df,2p) level, which has been proved to provide accurate energies in most cases. Unless noted, the CCSD(T) energies with inclusion of MP2 zero-point vibrational energy (ZPE) are used throughout. All calculations were carried out using the Gaussian 03 program packages [18]. 

According to the variational transition-state theory (VTST) and microcanonical RRKM [19] theory, the kinetic calculations for this multi-channel and multi-well reaction were carried out using the Multiwell 2011 program [20,21] on the basis of the PES obtained above in order to obtain the rate constants and the branching ratios for the key product channels. 

## 3. Results and Discussion

### 3.1. Potential Energy Surface and Reaction Mechanism

**Figure 1:**
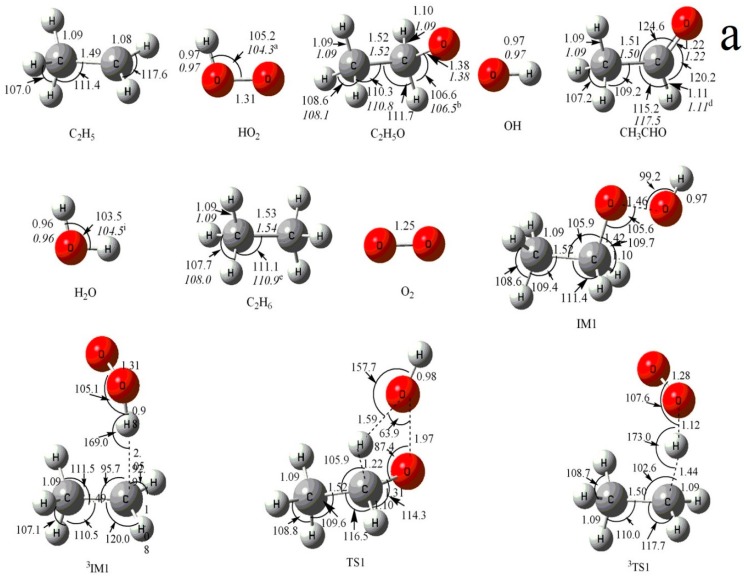

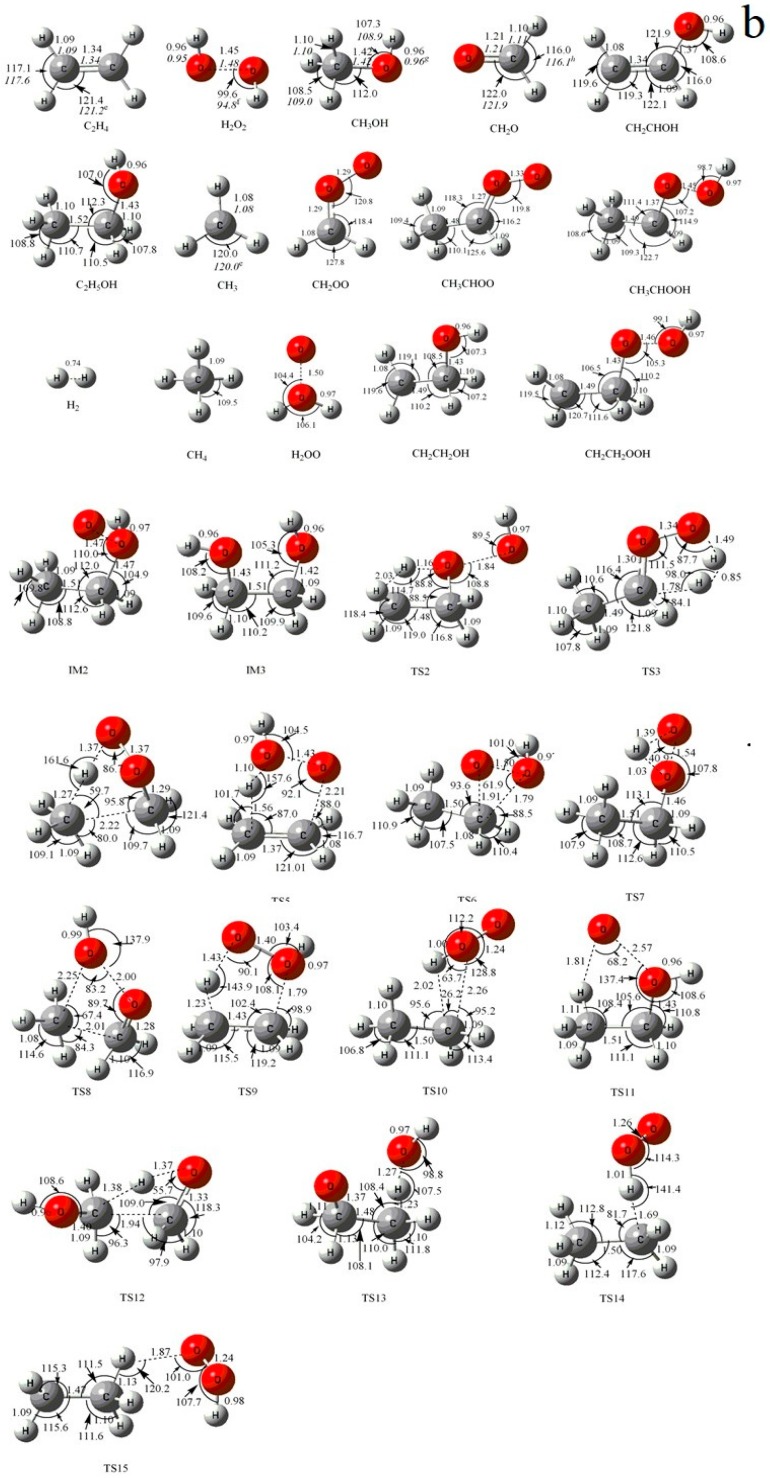
(**a**) MP2/6-311++G(d,p) optimized geometries for the reactants, products, intermediates (IM) and the corresponding transition states (TS) of the primary product channels of the C_2_H_5_ + HO_2_ reaction. (**b**) MP2/6-311++G(d,p) optimized geometries for the reactants, products, intermediates (IM) and the corresponding transition states (TS) of the secondary product channels of the C_2_H_5_ + HO_2_ reaction. The values in parentheses are the pertinent experimental data from the literature, and *a*–*i* represent refs. [17,18,22,23,24,25,26,27,28], respectively. Bond lengths are in Å, and bond angles are in degree.

**Figure 2:**
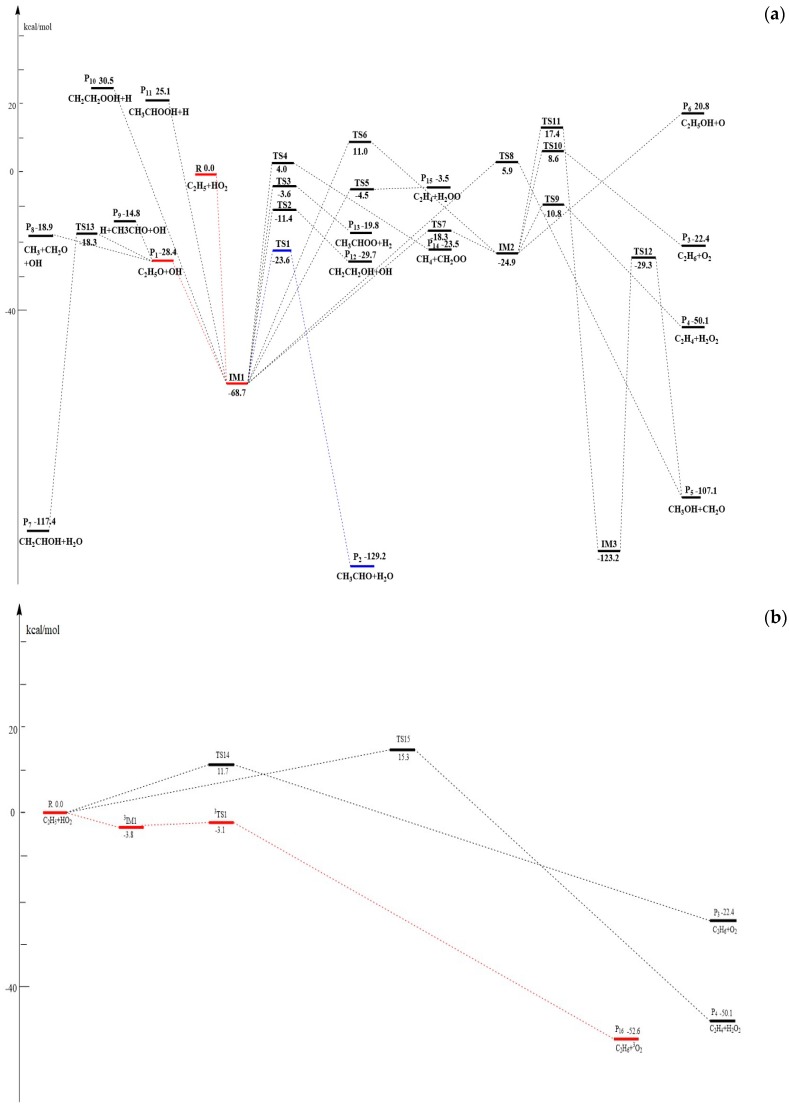
(**a**) Schematic singlet potential energy surface (PES) of the addition and dissociation reaction channels for the C_2_H_5_ + HO_2_ reaction at the CCSD(T)/6-311+G(3df,2p)//MP2/6-311++G(d,p)+ZPE level. (**b**) Schematic singlet and triplet PESs of the abstraction reaction channels for the C_2_H_5_ + HO_2_ reaction at the CCSD(T)/6-311+G(3df,2p)//MP2/6-311++G(d,p)+ZPE level.

The optimized geometries of the reactants, products, intermediates, and transition states for the C_2_H_5_ + HO_2_ reaction are shown in Figure 1a,b along with the available experimental data from the literature. It is seen that when comparison is available, the agreement between theoretical and experimental results is good, with the largest discrepancy within a factor of 2.0%. The schematic profiles of the singlet and triplet potential energy surfaces of the title reaction are depicted in Figure 2a,b. The total energy of the reactant **R** (C_2_H_5_ + HO_2_) is set to be zero for reference, and the values in parentheses are relative energies in kcal/mol with reference to **R**.

#### 3.1.1. Association and Decomposition Channels 

The reaction of HO_2_ with the C_2_H_5_ radical may proceed barrierlessly via the addition of the O-atom of HO_2_ to the C-atom of C_2_H_5_, leading to the energy-rich entrance intermediate **IM1** C_2_H_5_OOH (−68.7 kcal/mol) (see Figure 2a). The process is a characteristic feature of the typical radical–radical reaction mechanism. This process makes the intermediate **IM1** highly activated so that further isomerization or dissociation from it is possible.

Firstly, **IM1** can decompose by direct bond-breaking to form different products **P_1_** C_2_H_5_O + OH (−28.4 kcal/mol), **P_10_** CH_2_CH_2_OOH + H (30.5 kcal/mol) and **P_11_** CH_3_CHOOH + H (25.1 kcal/mol) with the breaking bonds of O–O, C_α_–H and C_β_–H, respectively. These processes occur via loose, variational transition states without any barriers. Since **P_10_** and **P_11_** have much less thermodynamic stability, the pathways of **P_10_** and **P_11_** formation are energetically unfeasible and could be neglected in the kinetic calculations. Product **P_1_** may have taken place in a secondary dissociation reaction leading to **P_7_** CH_2_CHOH + H_2_O (−117.4 kcal/mol) via a H-abstraction transition state TS13 (−18.3 kcal/mol), **P_8_** CH_3_ + CH_2_O + OH (−18.9 kcal/mol) via direct C–C bond rupture, and **P_9_** H + CH_3_CHO + OH (−14.8 kcal/mol) via direct H-extrusion. However, due to much higher energies and more steps required in the formations of **P_7_**, **P_8_**, and **P_9_**, these three secondary reaction channels do not need to be considered in the kinetic calculations. 

Secondly, starting from **IM1**, six kinds of other fragmentation pathways through tight transition states are also identified: (1) 1,3 H_2_O-elimination through a four-membered ring transition state TS1 (−23.6 kcal/mol) to form **P_2_** CH_3_CHO + H_2_O (−129.2 kcal/mol), (2) 1,3-OH migration through transition state TS8 (5.9 kcal/mol) to yield **P_5_** CH_3_OH + CH_2_O (−107.1 kcal/mol), (3) concerted 1,3 H-shift and OH-extrusion through four-membered ring transition state TS2 (−11.4 kcal/mol) to produce **P_12_** CH_2_CH_2_OH + OH (−29.7 kcal/mol), (4) 1,3 H_2_-elimination to yield **P_13_** CH_3_CHOO + H_2_ (−19.8 kcal/mol) via a five-membered ring transition state TS3 (−3.6 kcal/mol), (5) concerted 1,4-H-shift and C–C bond rupture process leading to **P_14_** CH_4_ + CH_2_OO (−23.5 kcal/mol) via TS4 (4.0 kcal/mol), and (6) concerted 1,4 H-migration and C-O bond fission process to yield **P_15_** C_2_H_4_ + H_2_OO (−3.5 kcal/mol) via TS5 (−4.5 kcal/mol). It is easily seen that among them, the most feasible channel is the formation of **P_2_**, while the other channels are negligible because the energies of TS8, TS2, TS3, TS4, and TS5 in channels (2)–(6) are much higher than TS1 in channel (1). 

In addition, **IM1** can also isomerize to intermediate **IM2** CH_3_CH_2_O(H)O (−24.9 kcal/mol) by 1,2 H-shift transition state TS7 (−18.3 kcal/mol) or a three-membered ring transition state TS6 (11.0 kcal/mol). Once isomer **IM2** is formed, four possible reaction pathways could take place: (a) a concerted 1,4 H-migration and C–O bond fission process to yield **P_4_** C_2_H_4_ + H_2_O_2_ (−50.1 kcal/mol) via TS9 (−10.8 kcal/mol), (b) dissociation to **P_3_** C_2_H_6_ + ^1^O_2_ (−22.4 kcal/mol) via a 1,2 H-shift transition state TS10 (8.6 kcal/mol), (c) O-extrusion of the O–O bond to form **P_6_** C_2_H_5_OH + O (20.8 kcal/mol) with no distinct barriers, and (d) O–H bond formation accompanied by C–H and O–O bond rupture to form **IM3** CH_2_(OH)CH_2_OH (−123.2 kcal/mol) via TS11 after surmounting a high energy barrier of 42.3 kcal/mol, then **IM3** can undergo a concerted 1,3 H-migration and C–C bond fission process to yield **P_5_** via TS12 (−29.3 kcal/mol). Clearly, these dissociation paths that start from **IM2** would make negligible contribution to the reaction, because of their much larger barriers.

#### 3.1.2. H-Abstraction Channels

As shown in Figure 2b, the HO_2_ can directly abstract the H atom from C_2_H_5_ via TS15 to form **P_4_** C_2_H_4_ + H_2_O_2_ (−50.1 kcal/mol), or C_2_H_5_ directly abstracts the H atom from HO_2_ via TS14 to produce **P_3_** C_2_H_6_ + ^1^O_2_ (−22.4 kcal/mol) on the singlet PES. The reaction barriers are 15.3 and 11.7 kcal/mol, respectively. Furthermore, the H-abstraction reaction can take place on a triplet PES. A van der Waals hydrogen-bonded complex, **^3^IM1** (−3.8 kcal/mol), is formed first at the entrance channel with the O-H bond distance of 2.05 Å, which is then followed by a hydrogen abstraction that fragments readily via ^3^TS1 to give **P_16_** C_2_H_6_ + ^3^O_2_ (−22.4 kcal/mol). This process needs to overcome a small barrier of just 0.7 kcal/mol. The reaction channels that form **P_3_** and **P_4_** are infeasible in the kinetics due to their high energy barriers.

In summary, on the singlet PES, the formations of one intermediate **IM1** as well as two primary product fragments **P_1_**, **P_2_** and one direct H-abstraction product **P_16_** through the triplet PES are most likely accessible in energy for the reaction of C_2_H_5_ + HO_2_ (see Figure 1 and Figure 2). Because the competition between the variety products under different temperatures and pressures cannot be determined solely on the PESs, VTST and RRKM calculations are performed to obtain the rate constants and branching ratios of all these competitive channels in the following section.

### 3.2. Kinetic Calculations

Product distributions for the key products are calculated based on the PES obtained above for the C_2_H_5_ + HO_2_ reaction using the MultiWell 2011 program [20,21] in the temperature range of 220 to 3000 K and in the pressure range of 1 × 10^−4^ to 100 bar. For the barrierless channels, such as the entrance channel for the formation of the chemically activated **IM1** C_2_H_5_OOH and the dissociation channel from **IM1** to **P_1_** C_2_H_5_O + OH, VTST [29,30] was used to locate the kinetic bottleneck. For this purpose, we carried out constrained optimizations at fixed C–O bond length or fixed O–O bond length in C_2_H_5_OOH at the multi-reference self-consistent field theory CASSCF(8,6)/aug-cc-pvdz level of theory. The total energies along the reaction coordinate were refined by single-point energy calculations using the CASPT2(8,6)/aug-cc-pvdz level. CASPT2//CAS calculations were done with the MOLPRO 2006 program [31,32]. The energetic and molecular parameters (reaction barriers, moment of inertia, and vibrational frequencies) of the reactants, intermediates and transition states from the ab initio calculations were used in kinetic calculations. Rate constants for direct H abstraction reactions, including the formation channels of **P_3_**, **P_4_**, and **P_16_**, were also obtained using the variation transition-state theory (CVT) with the small curvature tunneling (SCT) correction by means of the POLYRATE 9.7 program [33,34]. The total rate constant (*k_tot_*) is obtained as the sum of the individual rate constants associated with the corresponding channels. The calculated rate constant value, 6.88 × 10^−11^ cm^3^·molecule^−1^·s^−1^ at 293 K, is in good agreement with the experimental values, 4.98 × 10^−11^ and (5.15 ± 1.66) × 10^−11^ cm^3^·molecule^−1^·s^−1^.

**Figure 3:**
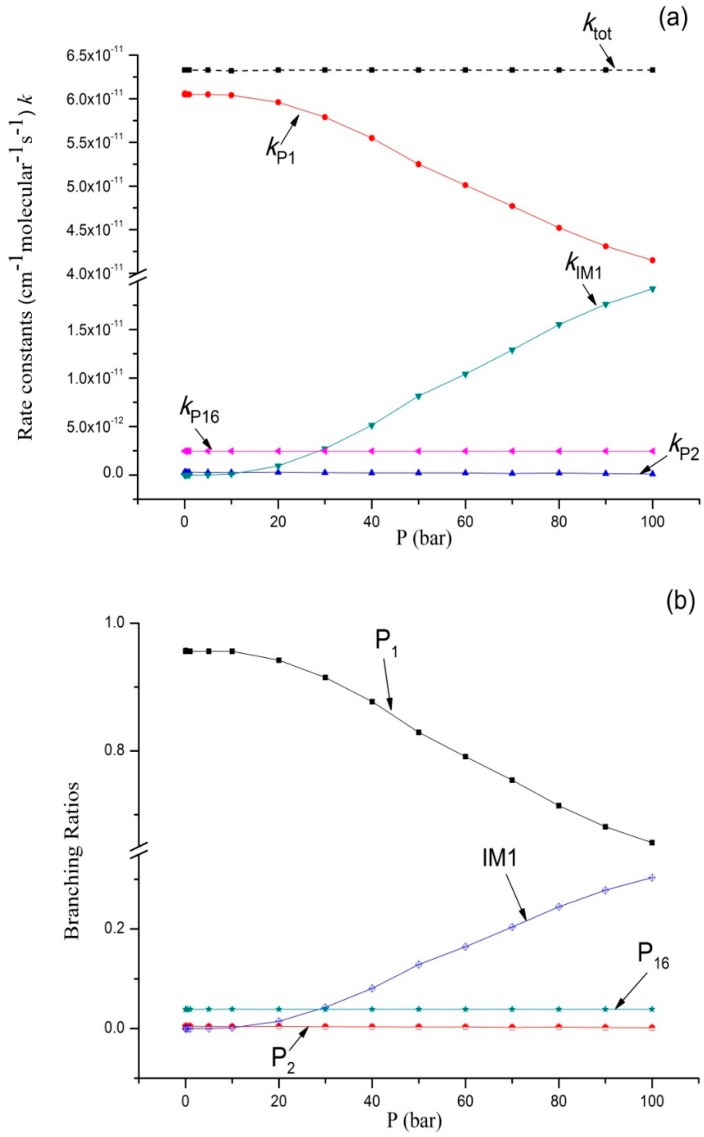
(**a**) Rate constants of total and various reaction channels at 293 K in a pressure range from 1 × 10^−4^ bar to 100 bar; (**b**) Branching ratios of various reaction channels at 293 K in a pressure range from 1 × 10^−4^ bar to 100 bar.

Variations of the reaction rate constants and branching ratios of the reaction channels at 293K when pressure increases from 1 × 10^−^^4^ bar–100 bar are shown in Figure 3a,b. *k_tot_* seemed independent from pressure. The dissociation bimolecular product **P_1_** C_2_H_5_O + OH was the major product, which is negatively correlated with pressure. The output of **P_1_** decreased from 0.96 at 1 × 10^−^^4^ bar to 0.66 at 100 bar. It is interesting that *k*_P16_ and *k*_P2_ were predicted independently from pressure, and *k*_P16_ > *k*_P2_. The branching ratio of *k*_P16_ reached 0.04 throughout the whole pressure range. The contribution of **P_2_** generation to the total reaction rate can be ignored. *k_IM_*_1_ increases with an increase in pressure (>10 bar), and the output of **IM1** C_2_H_5_OOH increased continuously and reached 0.31 at 100 bar. These computational results were compared with the available experimental data; our findings showed that **P_1_** C_2_H_5_O + OH was the major product. According to the theoretical computation, bimolecular products **P_2_** CH_3_CHO + H_2_O and **P_16_** C_2_H_6_ + ^3^O_2_, as well as unimolecular product **IM1** C_2_H_5_OOH, had small outputs. This is why they were difficult to detect in this experiment.

**Figure 4:**
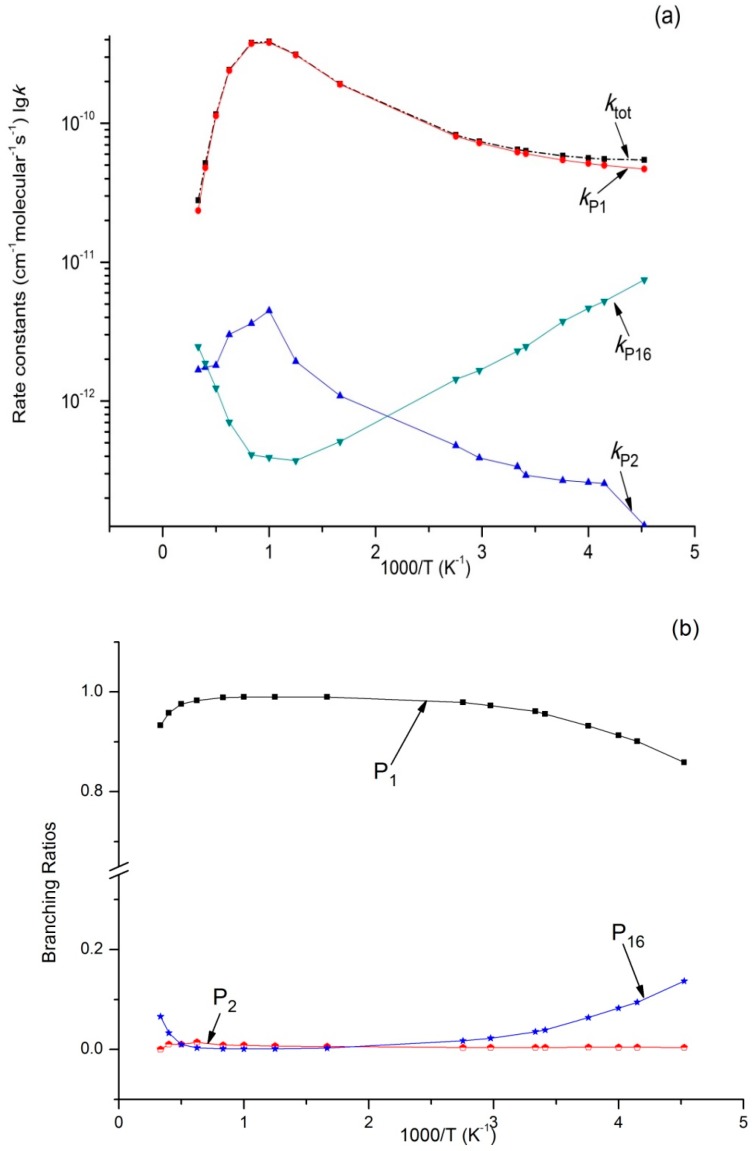
(**a**) Rate constants of total (*k_tot_*) and various reaction channels (*k*_P1_, *k*_P2_, *k*_P16_, and *k*_IM1_) at 1 × 10^−4^ Bar in a temperature range of 220 K to 3000 K; (**b**) Branching ratios of various reaction channels at 1 × 10^−4^ Bar in a temperature range of 220 K to 3000 K.

**Figure 5:**
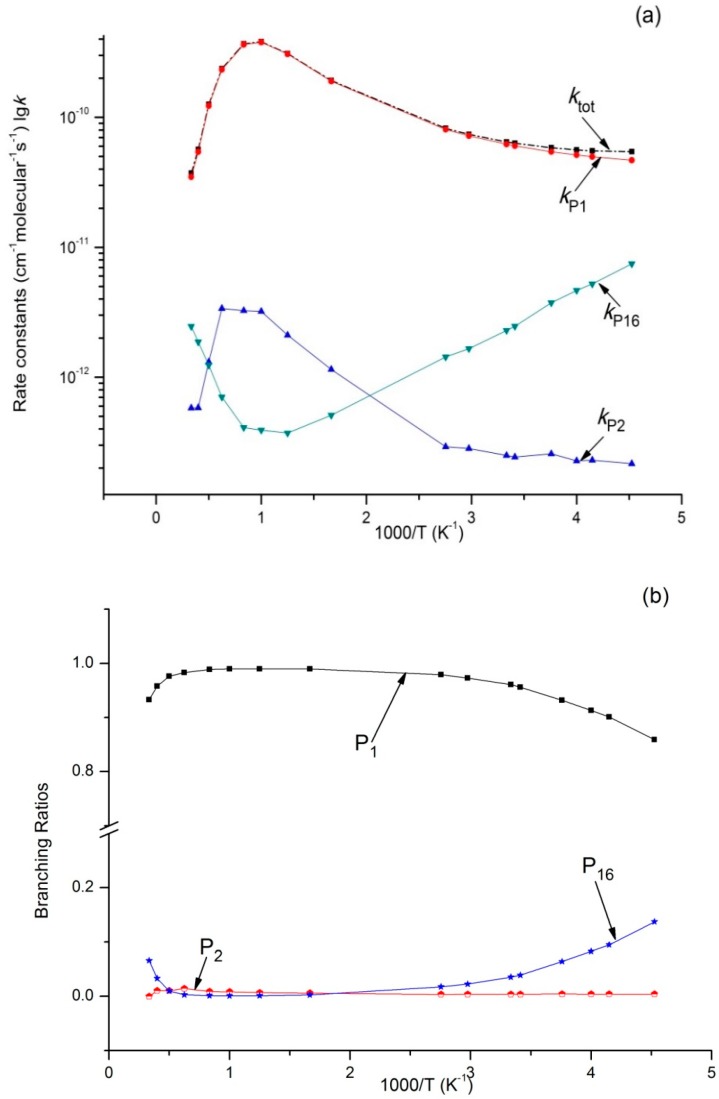
(**a**) Rate constants of total (*k_tot_*) and various reaction channels (*k*_P1_, *k*_P2_, *k*_P16_, and *k*_IM1_) at 1 bar in a temperature range of 220 K to 3000 K; (**b**) Branching ratios of various reaction channels at 1 bar in a temperature range of 220 K to 3000 K.

**Figure 6:**
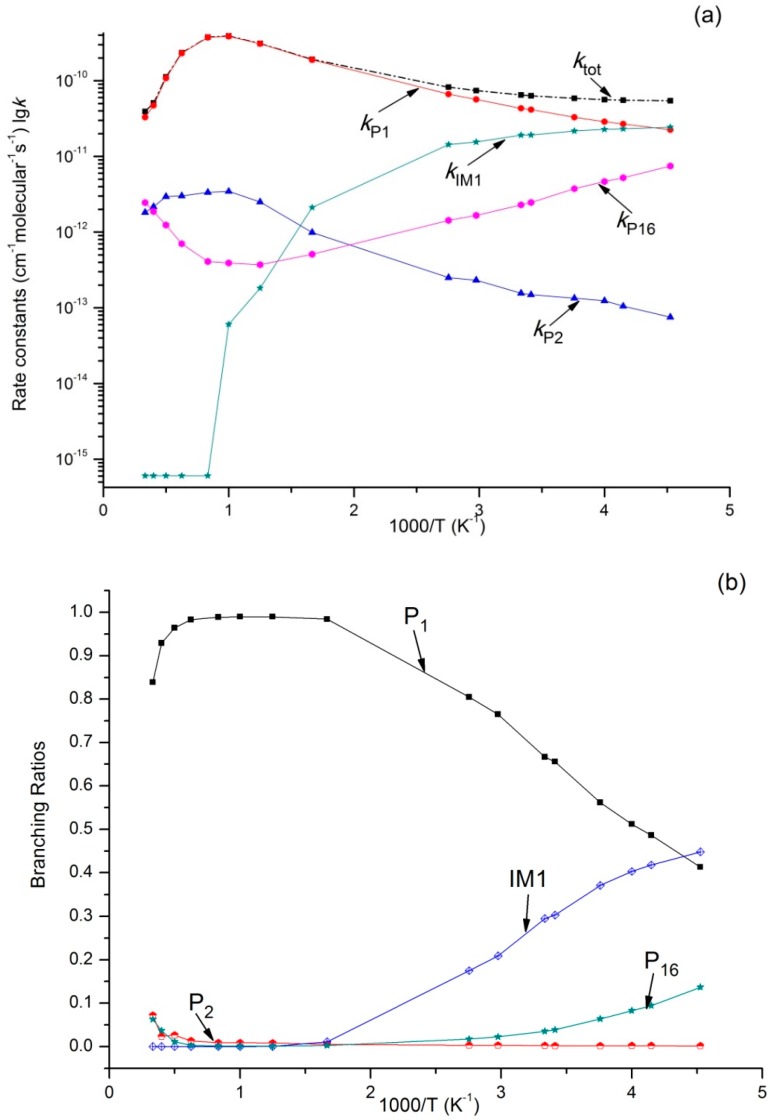
(**a**) Rate constants of total (*k_tot_*) and various reaction channels (*k*_P1_, *k*_P2_, *k*_P16_, and *k*_IM1_) at 100 bar in a temperature range of 220 K to 3000 K; (**b**) Branching ratios of various reaction channels at 100 bar in a temperature range of 220 K to 3000 K.

Variations of *k_tot_* as well as reaction rate constants and branching ratios of reaction channels at 1 × 10^−4^ bar, 1 bar, and 100 bar when the temperature increases from 220–3000 K are presented in Figure 4a,b, Figure 5a,b, and Figure 6a,b. At the very beginning, *k_tot_* increased from 5.45 × 10^−11^ cm^3^·molecule^−1^·s^−1^ at 220 K to 3.87 × 10^−10^ cm^3^·molecule^−1^·s^−1^ at 1000 K, but then decreased to 3.73 × 10^−11^ cm^3^·molecule^−1^·s^−1^ at 3000 K. Outputs of bimolecular products **P_1_** and **P_2_** showed similar temperature dependence with *k_tot_*. However, the output of **P_16_** showed the opposite, firstly decreasing to 3.72 × 10^−13^ cm^3^·molecule^−1^·s^−1^ at 800 K and then increasing to 2.46 × 10^−12^ cm^3^·molecule^−1^·s^−1^ at 3000 K. Meanwhile, *k*_IM1_ was negatively correlated with temperature under high pressure at 100 bar. According to Figure 4b and Figure 5b, the reaction mainly produced dissociation products under 1 × 10^−4^ bar and 1 bar, while the impact stabilization effect of the intermediates was neglected. The bimolecular product **P_1_** was the main product. Its output increased from 0.86 at 220 K to 0.99 at 1000 K and then decreased to 0.93 (1 × 10^−4^ bar) and 0.84 (1 bar) at 3000 K. Under 1 × 10^−4^ bar and 1 bar, the maximum branching ratio of secondary product **P_16_** at 220 K was 0.14 and reached the peak (0.07) at 300 K. Under high pressure (100 bar) (Figure 6b), output of **P_1_** increased quickly with the temperature rise and reached the peak (0.99) at 1000 K. **P_1_** became the major product after the temperature reached 227 K. However, the intermediate stabilized by impact (**IM1**) was the major product under low temperature, while other products like **P_2_** and **P_16_** had a very small branching ratio within the whole studying temperature range (≤0.1). According to our kinetic calculation of the C_2_H_5_ + HOO reaction, the effect of pressure on product distribution declined after temperatures exceeded 293 K and the dissociation product **P_1_** C_2_H_5_O + OH became the major product. In other words, under low temperature, relative output showed strong pressure dependence, and the stabilization effect of **IM1** C_2_H_5_OOH became increasingly important as pressure increased.

**Figure 7:**
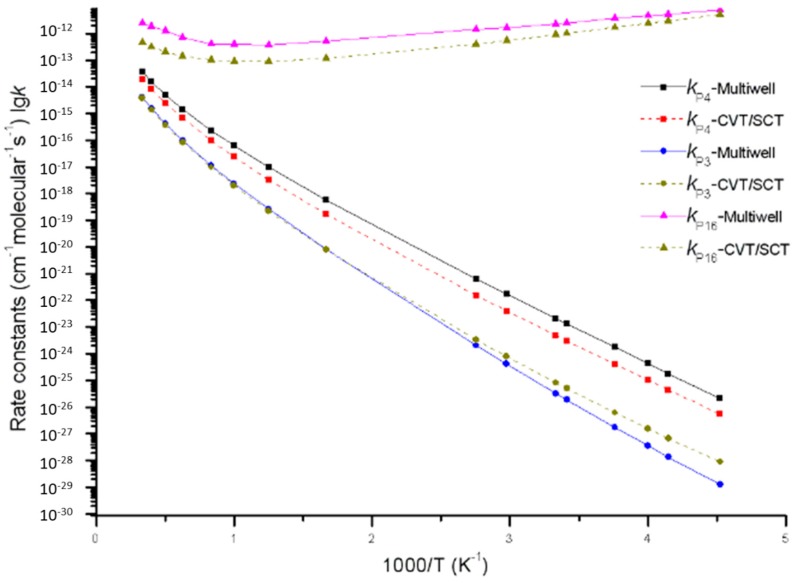
Rate constants of direct H-abstraction reaction channels (including generation channels of **P_3_**, **P_4_** and **P_16_**) calculated by using the MultiWell program and the Polyrate program, respectively.

Furthermore, it is very important to compare the MultiWell program results of direct H-abstraction reaction channels (including generation channels of **P_3_** and **P_4_** on a singlet potential energy surface and generation channel of **P_16_** on a triplet potential energy surface) with the Polyrate predicted results. Reaction rate constants calculated by the MultiWell program and the Polyrate program are shown in Figure 7. Under low temperature, the MultiWell and Polyrate programs report similar rate constants of triplet product **P_16_** C_2_H_6_ + ^3^O_2_. Under high temperature, they conformed to each other more in terms of singlet product **P_3_** C_2_H_6_ + ^1^O_2_ and **P_4_** C_2_H_4_ + H_2_O_2_. At 220 K, *k*_P3_, *k*_P4_, and *k*_P16_ calculated by the MultiWell program were 0.14, 3.3, and 1.4 times higher than those calculated by the CVT/SCT model. At 3000 K, *k*_P3_, *k*_P4_, and *k*_P16_ calculated by the MultiWell program were 1.1, 2, and 5 times more satisfactory than those calculated by the CVT/SCT model. This paper concludes that under low energy barrier and low temperature range (or high energy barrier and high temperature range), reaction rate constants calculated by both the MultiWell and Polyrate programs have high accuracy.

## 4. Conclusions

In this paper, singlet and triplet potential energy surfaces of the C_2_H_5_ + HOO reaction were computed on the CCSD(T)/6-311+G(3df,2p)//MP2/6-311++G(d,p) theoretical level. Rate constants and branching ratios of major reaction channels under the temperature range 220 K–3000 K and pressure range 1 × 10^−4^ bar–100 bar were predicted. According to the computational results, the C_2_H_5_ + HOO reaction occurred through addition and abstraction on the singlet potential energy surface. In other words, the O atom in HOO attacked the C atom in C_2_H_5_ to form an entrance intermediate with rich energy **IM1** C_2_H_5_OOH through an energy barrier-free addition reaction. Starting from **IM1**, direct energy barrier-free O–O bond breakage generated the major product **P_1_** C_2_H_5_O + OH, and **P_2_** CH_3_CHO + H_2_O was the secondary product. Alternatively, HOO can directly abstract a H atom in C_2_H_5_ to produce **P_4_** C_2_H_4_ + H_2_O_2_, and C_2_H_5_ can also directly abstract a H atom in HOO to produce **P_3_** C_2_H_6_ + ^1^O_2_. These two H-abstraction reaction channels have to overcome high energy barriers and are unfeasible in kinetics. On the triplet potential energy surface, generation of **P_16_** C_2_H_6_ + ^3^O_2_ through a H-abstraction reaction has to pass through a loose van der Waals complex, **^3^IM1** ^3^C_2_H_5_HOO, and overcome a small energy barrier. The kinetic calculation demonstrates that the impact-stabilized intermediate **IM1** is the major product under high pressure and low temperature, but the bimolecular product **P_1_** is the major product under low pressure or high temperature. Under 293 K, **P_1_** was the major product within the studied pressure range, which conformed well to experimental prediction. The branching ratio of **P_1_** output was inversely proportional to pressure, decreasing from 0.96 at 1 × 10^−4^ bar to 0.66 at 100 bar. **IM1** became the secondary product after pressure exceeded 30 bar (the maximum output is 0.31). Research results in this paper are conducive to deepening our understanding on the mechanisms and kinetics of the C_2_H_5_ + HOO reaction and determining its possible products.
